# Comprehensive Eutrophication Assessment Based on Fuzzy Matter Element Model and Monte Carlo-Triangular Fuzzy Numbers Approach

**DOI:** 10.3390/ijerph16101769

**Published:** 2019-05-19

**Authors:** Yumin Wang, Weijian Ran

**Affiliations:** 1 Department of Energy and Environment, Southeast University, Nanjing 210096, China; 2 School of Glasgow, University of Electronic Science and Technology, Chengdu 610054, China; 2017200504014@std.uestc.edu.cn

**Keywords:** triangle fuzzy number, Monte Carlo approach, fuzzy matter element model, eutrophication evaluation

## Abstract

Evaluating the eutrophication level of lakes with a single method alone is challenging since uncertain, fuzzy, and complex processes exist in eutrophication evaluations. The parameters selected for assessing eutrophication include chlorophyII-a, chemical oxygen demand, total phosphorus, total nitrogen, and clarity. Firstly, to deal with the uncertainties and fuzziness of data, triangular fuzzy numbers (TFN) were applied to describe the fuzziness of parameters. Secondly, to assess the eutrophication grade of lakes comprehensively, an improved fuzzy matter element (FME) approach was incorporated with TFNs with weights determined by combination of entropy method and analytic hierarchy process (AHP). In addition, the Monte Carlo (MC) approach was applied to easily simulate the arithmetic operations of eutrophication evaluation. The hybrid model of TFN, FME, and MC method is termed as the TFN–MC–FME model, which can provide more valuable information for decision makers. The developed model was applied to assess the eutrophication levels of 24 typical lakes in China. The evaluation indicators were expressed by TFNs input into the FME model to evaluate eutrophication grade. The results of MC simulation supplied quantitative information of possible intervals, the corresponding probabilities, as well as the comprehensive eutrophication levels. The eutrophication grades obtained for most lakes were identical to the results of the other three methods, which proved the correctness of the model. The presented methodology can be employed to process the data uncertainties and fuzziness by stochastically simulating their distribution characteristics, and obtain a better understanding of eutrophication levels. Moreover, the proposed model can also describe the trend of eutrophication development in lakes, and provide more valuable information for lake management authorities.

## 1. Introduction

Water resources of desirable quantity and suitable quality are a prerequisite for sustainable development of the economy, society and ecology [1]. In China, most lakes have serious water environmental problems, such as shrinkage of water surface area, eutrophication of lakes, organic pollution, etc. Among them, eutrophication due to natural process (e.g., weathering, precipitation, soil erosion, etc.) and anthropogenic activities (industrial pollution, domestic drainage, etc.), has become a serious and common concern which threatens the public health and the ecological environment, even inducing water-borne diseases [2,3,4,5,6].

Therefore, it is significant to understand lakes’ eutrophication conditions by assessing their eutrophication level scientifically and objectively. In previous studies, various mathematical approaches, listed in Table 1, were applied to evaluate the eutrophication of lakes and reservoirs [5,7,8,9,10,11].

Since the methods applied have their advantages and limitations, to avoid the disadvantages as well as develop the advantages, it is necessary to combine multifarious methods so as to improve the assessment process. Indicators used during the process of eutrophication assessment include chlorophyII-a (Chl-a), chemical oxygen demand (COD_Mn_), total phosphorus (TP), total nitrogen (TN), and clarity (SD). When indicator monitoring data are scarce, imprecise, varied temporally and spatially with wide variation intervals, the input data for assessing the eutrophication status are not determinant but rather have characteristics of uncertainty and inaccuracy. To solve the uncertainty and inaccuracy of input data, fuzzy set theory had been employed, such as triangular fuzzy numbers (TFN) [12]. The TFN approach is suitable for processing uncertain data with a triangular distribution. Generally speaking, the concentrations of indicators obey a Gaussian distribution or an approximate Gaussian distribution. However, if the input data are scarce and have a wide range, the Gaussian distribution can be replaced by a triangular distribution [13]. In addition, since contradictory problems exist in eutrophication evaluation, for example, some indicators belong to eutrophication grade A, while other indicators belong to eutrophication grade B, this increases the difficulty in determining the eutrophication grade of a specific lake. To deal with the problem, the fuzzy matter element (FME) model was proposed, which is suitable for multi-factor assessment based on fuzzy mathematics theory [14]. The fuzzy matter element model is an approach that combines fuzzy theory and a matter element model, which takes the advantages of both fuzzy theory and matter element theory, and had been widely used for assessments [3,8,15,16]. To deal with the data uncertainty and contradiction problems in eutrophication assessment, a FME model with the input data expressed by TFNs was employed in this paper, which is termed the TFN–FME method. However, when the indicators are expressed by various TFNs, the arithmetic and function operations among them in the FME model will result in a complex assessment process, and generate more uncertain information [17,18]. Therefore, the Monte Carlo (MC) approach is applied to solve the complex operation of TFNs by converting the arithmetic operations into real number operations, which can better solve the uncertainty problem existing in data so as to simplify the operation process. Furthermore, a hybrid model termed as TFN–MC–FME was proposed, which can approximate a Gaussian distribution of evaluation indicators, deal with complex operations of TFNs, and avoid the contradiction problems. The proposed method integrates together all the advantages of the TFN, MC, and FME methods, and was applied to evaluate the eutrophication levels of 24 typical lakes in China.

In this paper, the TFN method was applied to deal with the uncertainty of monitored data information, and the FME method was proposed to solve the contradiction problems in the process of eutrophication assessment. In addition, MC simulation was employed to solve the complex calculations of TFN, which resulted in the development of the proposed TFN–MC–FME model. The developed hybrid model was applied to 24 typical lakes and reservoirs in China and the validity of the proposed model was assessed through comparisons with three other methods. Finally, the probabilistic eutrophication levels of lakes were obtained, and valuable information was supplied for environmental management agency to propose and implement reasonable policies to control and prevent eutrophication.

## 2. Methodology

### 2.1. Triangular Fuzzy Numbers (TFN) Approach

The uncertain information of monitoring data can be solved by TFN method according to the variation intervals considering the average values $\bar{A}$ and standard deviations σ of the data. The detail of the TFN approach was explained as follows:

Suppose *A*_1_, *A*_2_, and *A*_3_ are the lower, expected and upper value of a fuzzy variable, respectively, with *A*_1_ < *A*_2_ < *A*_3_. The TFN can be expressed by $\tilde{A}=(A_{1},A_{2},A_{3})$. The triangular probabilistic distribution of $\tilde{A}$ can be defined by Equation (1) as follows:

(1) $$\tilde{A}=\{\begin{array}{cc} 0 & x\leq A_{1}\\ \left(x-A_{1})/(A_{2}-A_{1}\right) & A_{1}<x\leq A_{2}\\ \left(A_{3}-x)/(A_{3}-A_{2}\right) & A_{2}<x\leq A_{3}\\ 0 & x>A_{3} \end{array}$$

The TFN $\tilde{A}$ was defined by Equation (2) as follows [12]:

(2) $$A_{1}=\max(\min A,\bar{A}-2\sigma ),\;A_{2}=\bar{A},\;A_{3}=\min(\bar{A}+2\sigma ,\max A)$$

where $\bar{A}$ is the average value of the data, and σ is the standard deviation of the data.

### 2.2. Monte Carlo (MC) Approach

The operation of uncertain variables with triangular distribution was performed by the MC approach. By generating a series of sample data based on the triangular distribution of each variable, the MC method can transform the uncertain data expressed by a distribution function into stochastic numbers, and can perform arithmetic operations easily. Therefore, the MC method can solve the difficulty of multi-variables’ mathematical operations in the FME model described in detail in Section 2.3. The MC method was performed by theCrystal Ball software, which is applied as an analytical tool to help execute, analyze, and make decisions by performing simulations and forecasting of data on spreadsheet models [18]. It can generate random series of possible values based on the probability distribution type of variables with settled operation parameters. By running the model, the probability distribution of predictive variables is obtained. In addition, by setting intervals, the corresponding probabilities of predictive variables in the set intervals can also be obtained.

### 2.3. Improved Fuzzy Matter Element Model

The process of performing improved fuzzy matter element model is described as follows:

#### 2.3.1. Establish the Fuzzy Matter Element model of Eutrophication Assessment

The fuzzy matter element model is consisted of a triple ordered matrix of “objects, characteristics, and fuzzy values”, which were denoted as *U* = (*C*, *G*, *μ*), expressed as by Equation (3) as follows:

(3) $$U_{mn}^{k}=\left[\begin{array}{ccccc} & G_{1} & G_{2} & \cdots & G_{n}\\ C_{1} & \mu_{11}^{k} & \mu_{12}^{k} & \cdots & \mu_{13}^{k}\\ C_{2} & \mu_{21}^{k} & \mu_{22}^{k} & \cdots & \mu_{23}^{k}\\ \vdots & \vdots & \vdots & \vdots & \vdots \\ C_{m} & \mu_{m1}^{k} & \mu_{m2}^{k} & \cdots & \mu_{mn}^{k} \end{array}\right]$$

where $U_{mn}^{k}$ is fuzzy matter element matrix for the *k*-th studied object (lake), C*_i_* is the *i*-th indicator, *i* = 1, 2, …, *m*; G*_j_* is the *j*-th eutrophication grade, *j* = 1, 2, …, *n*; $\mu_{ij}^{k}$ is the fuzzy membership degree of the *i*-th indicator to the *j*-th grade, which was calculated based on fuzzy membership functions and the corresponding eutrophication classifications. The normal membership functions were adopted in this paper since normal distributions of membership functions were adopted in numerous observations [15,19], expressed by Equation (4) as follows:

(4) $$\mu_{ij}^{k}=\exp[-{\left(\frac{x_{i}^{k}-a_{ij}}{b_{ij}}\right)}^{2}]$$

where $\mu_{ij}^{k}$ is fuzzy the membership function of the *i*-th indicator to the *j*-th classification criterion for the *k*-th lake, which is calculated by MC method, $x_{i}^{k}$ is the measured concentration of the *i*-th indicator in the *k*-th lake, which is expressed by variables with triangular distribution, i.e., TFNs expressed by $\tilde{A}$, *a_ij_* and *b_ij_* are the constants with the constraints of $a_{ij}>0$, and $b_{ij}>0$. The definitions of *a_ij_* and *b_ij_* should satisfy conditions as follows: (1) for cost indicators (i.e., the smaller, the better), such as Chl-a, COD_Mn_, TN, and TP, when $x_{i}^{k}$ is equal to the lower boundary in grade I, $\mu_{i1}^{k}=1.0$; when $x_{i}^{k}$ is greater than or equal to the upper boundary in grade VI in Table 2, $\mu_{i6}^{k}=1.0$; (2) for efficiency indicators (i.e., the larger, the better), such as SD, when $x_{i}^{k}$ is greater than or equal to upper boundary in grade I, $\mu_{i1}^{k}=1.0$; when $x_{i}^{k}$ is equal to lower boundary in grade VI, $\mu_{i6}^{k}=1.0$; (3) in grade II, III, IV, and V, when $x_{i}^{k}$ is the average value of the *j*-th grade criterion of the *i*-th indicator for the *k*-th lake, $\mu_{ij}^{k}=1.0$. Therefore, the definitions of *a_ij_* and *b_ij_* were expressed by Equations (5) and (6) as follows:

(5) $$a_{ij}=\{\begin{array}{cc} x_{l}(x_{u}), & j=1\\ \frac{x_{l}+x_{u}}{2}, & j=2,3,4,5\\ x_{u}(x_{l)}, & j=6 \end{array}$$

(6) $$b_{ij}=\{\begin{array}{cc} \frac{x_{u}-x_{l}}{\sqrt{\ln2}}, & j=1\\ \frac{x_{u}-x_{l}}{2\sqrt{\ln2}}, & j=2,3,4,5\\ \frac{x_{u}-x_{l}}{\sqrt{\ln2}}, & j=6 \end{array}$$

where *x_l_* and *x_u_* are the lower and upper boundary values of the *j*-th classification criterion of the *i*-th indicator, respectively. In Equation (5), the definitions in the parentheses are for the efficiency indicators. 

Since each element in a row is the membership of each grade, the sum of each row should be equal to 1. Therefore, each element of each line in the fuzzy matter element matrix was normalized according to Equation (7) as follows:

(7) $$r_{ij}^{k}=\frac{\mu_{ij}^{k}}{\sum_{j=1}^{n}\mu_{ij}^{k}}$$

where $r_{ij}^{k}$ is the normalized fuzzy membership degree of the *i*-th indicator to the *j*-th grade for the *k*-th lake. The normalized fuzzy matrix is formed in terms of $R_{mn}^{k}$, shown in Equation (8) as follows:

(8) $$R_{mn}^{k}=\left[\begin{array}{ccccc} & G_{1} & G_{2} & \cdots & G_{n}\\ C_{1} & r_{11} & r_{12} & \cdots & r_{1n}\\ C_{2} & r_{21} & r_{22} & \cdots & r_{2n}\\ \vdots & \vdots & \vdots & \vdots & \vdots \\ C_{m} & r_{m1} & r_{m2} & \cdots & r_{mn} \end{array}\right]$$

where $R_{mn}^{k}$ is the normalized fuzzy matter element matrix for the *k*-th studied lake.

The upper boundary as well as lower boundary for each eutrophication grade in Equations (5) and (6) was determined by the criteria of the characteristic indicators listed in Table 2 [20]. 

**Figure 1:**
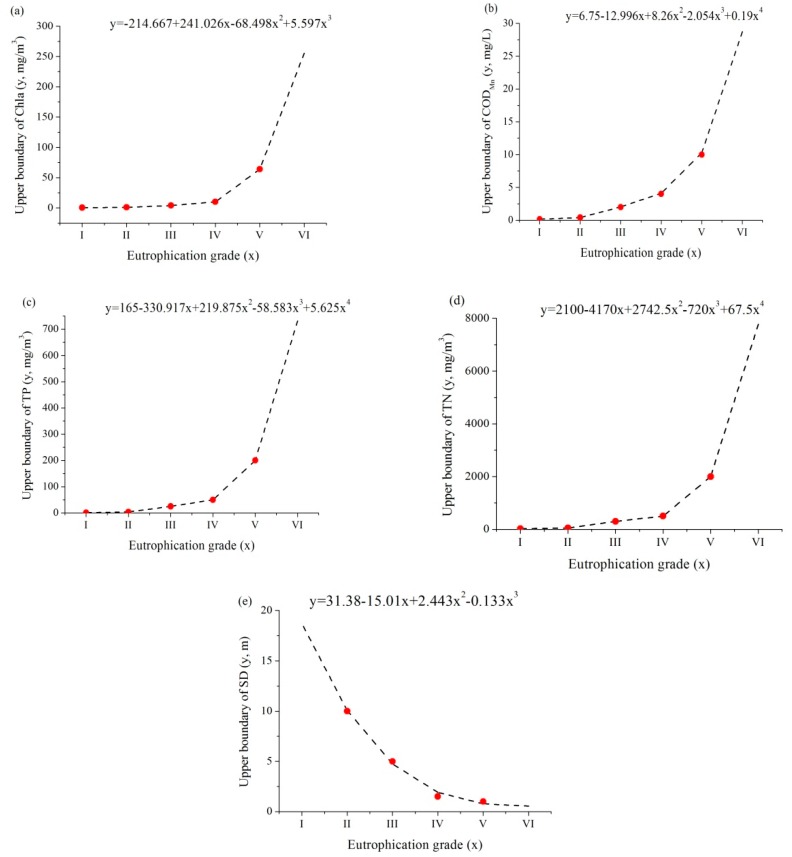
Nonlinear regression of the upper boundaries for (**a**) chlorophyII-a (Chl-a), (**b**) total phosphorus (TP), (**c**) total nitrogen (TN), (**d**) chemical oxygen demand (COD_Mn_), and (**e**) clarity (SD).

Since the upper boundaries of cost indicators (Chl-a, COD_Mn_, TP, and TN) for grade VI, as well as upper boundary of efficiency indicator (SD) for grade I are not available in Table 2, nonlinear regression analysis was performed with the assumption of upper boundary values increases with the grade to attain the pseudo-boundaries. The results are shown in Figure 1. 

#### 2.3.2. Determination of Weights

In the fuzzy matter element model, the weights of indicators were determined mainly by two categories of methods: subjective methods (e.g., adjacent indicator comparison, efficiency coefficient method, and analytical hierarchy process (AHP)), and objective weights (e.g., principal component analysis, factor analysis, variance coefficient approach, and entropy method [21]). Neither of them can describe the matter element integrally and systematically. Therefore, in this paper, the widely used AHP method for multi-criteria analysis and the entropy method were combined to calculate the weights of indicators in fuzzy matter element model for evaluating eutrophication, which can balance the potential subjective uncertainty of the AHP approach.

The term “entropy” originated from modern information theory, and regarded as a measure of disorder or uncertainty of a system [5]. It has been widely applied in the fields of uncertainty assessment. The entropy of the indicators can be calculated by Equation (9) as follows:

(9) $$H_{i}=-\sum_{k=1}^{K}p_{i}^{k}\cdot \ln p_{i}^{k}$$

where *H_i_* refers to the entropy of the *i*-th indicator, $p_{i}^{k}$ is the ratio between $y_{i}^{k}$ and sum of all the values for $y_{i}^{k}$, $p_{i}^{k}=\frac{y_{i}^{k}}{\sum_{k=1}^{K}y_{i}^{k}}$, where $y_{i}^{k}$ is the normalized data of the average value of *i*-th indicator for the *k*-th lake, and if $p_{i}^{k}$ = 0 then $\ln p_{i}^{k}$ = 0. The normalized data were calculated by Equations (10) and (11) for cost indicators and efficiency indicators, respectively:

(10) $$y_{i}^{k}=\frac{\underset{k}{\max}(x_{i}^{k})-x_{i}^{k}}{\underset{k}{\max}(x_{i}^{k})-\underset{k}{\min}(x_{i}^{k})}$$

(11) $$y_{i}^{k}=\frac{x_{i}^{k}-\underset{k}{\min}(x_{i}^{k})}{\underset{k}{\max}(x_{i}^{k})-\underset{k}{\min}(x_{i}^{k})}$$

Then weights of indicators based on entropy of the *i*-th criterion $w_{i}^{\prime }$ can be calculated by Equation (12) as follows:

(12) $$w_{i}^{\prime }=\frac{1-H_{i}}{m-\sum_{i=1}^{m}H_{i}}$$

where *m* is the number of indicators.

The entropy indicates the relative importance of indicators. The indicator with lower entropy corresponds to lower weights compared to other indicators. For balancing the potential subjective uncertainty of indicators, the AHP method was combined with entropy technology, given by Equation (13) as follows:

(13) $$w_{i}=\frac{r_{i}w_{i}^{\prime }}{\sum_{i=1}^{m}r_{i}w_{i}^{\prime }}$$

where $w_{i}$ is the weight determined by hybrid entropy–AHP method, $r_{i}$ is the weight determined by AHP method. Results are shown in Table 3.

#### 2.3.3. Calculation of Fuzzy Neartude

Fuzzy neartude (or fuzzy closeness degree) is the proximity between evaluated and standard samples, which is measured by introducing a similarity measure [8,11]. The greater fuzzy neartude to a certain eutrophication level indicates that the evaluated sample is closer to the eutrophication level. 

The fuzzy neartude of the *k*-th evaluated object to the *j*-th eutrophication level $\rho H_{j}^{k}$ is represented by Hamming neartude (*ρ*H) expressed by Equation (14) as follows:

(14) $$\rho H_{j}^{k}=1-\sum_{j=1}^{n}w_{i}\left|r_{ij}^{k}-r_{i0}\right|$$

where $r_{ij}^{k}$ is the element of normalized fuzzy matter element matrix, and $r_{i0}$ is the element of the ideal normalized fuzzy matter element matrix, expressed by Equation (15) as follows:

(15) $$R_{i0}=\left|\begin{array}{c} r_{10}\\ r_{20}\\ \vdots \\ r_{m0} \end{array}\right|=\left|\begin{array}{c} 1\\ 1\\ \vdots \\ 1 \end{array}\right|$$

where $R_{i0}$ is the ideal normalized fuzzy matter element matrix.

#### 2.3.4. Determine the Eutrophication Grade of Lakes

The eutrophication levels of lakes can be determined according to the non-integral feature value generated by Equation (16) as follows:

(16) $$J^{k}=\sum_{j=1}^{n}\frac{j\times \rho H_{j}}{\sum_{j=1}^{n}\rho H_{j}}$$

where $J^{k}$ is the non-integral eutrophication feature value of the *k*-th evaluated lake. The lower value of $J^{k}$ means better eutrophication grade and vice versa. The eutrophication grade of the *k*-th lake was defined according to classification in Table 4.

### 2.4. Comprehensive Eutrophication Evaluation Based on the TFN–MC–FME Model

The FME model can evaluate the eutrophication grade of lakes, but cannot be utilized to analyze the uncertainty of observed data for eutrophication evaluation, which can be solved by TFN approach that expresses data with an interval instead of a real number. Therefore, the combination of the FME model with the TFN approach can successfully solve the problem of the uncertainty of data. However, the calculation of uncertainty information expressed with different FME models is difficult to perform. Therefore, the MC approach is employed by converting the TFN numbers into real numbers to stochastically simulate the observed data to obtain the probabilistic results, which resolves the limitation of the TFN method. 

The specific operation processes of hybrid TFN–MC–FME model in the Crystal Ball software for assessing eutrophication grade are described as follows:

(1) In terms of the data processing method of TFN, the variables of Chl-a, COD_Mn_, TP, TN, and SD in the FME model were obtained by Equation (1), and expressed with $\tilde{A}$, such as $\tilde{A}=(A_{1},A_{2},A_{3})$.
(2) The above variables were defined as the independent variables, and probability distributions of the actual observed data were set as TFN distribution type in the Crystal Ball software. The *A*_1_, *A*_2_, and *A*_3_ of variables were typed in each data cell.
(3) According to Equations (3)–(8), the normalized fuzzy matter element matrixs for the *k*-th studied lake $R_{mn}^{k}$ were established. The weights of indictors were identified by Equations (9)–(13). Then, the fuzzy neartudes $\rho H_{j}^{k}$ of the *k*-th lake to the *j*-th grade were calculated by Equations (14) and (15). The eutrophication grades of lakes $J^{k}$ were determined by Equation (16).
(4) By running simulations in the Crystal Ball software, the corresponding probabilistic eutrophication grade for the *k*-th lake was obtained by setting the interval values of each eutrophication grade in the software. By further combining the probabilities with the eutrophication grade, the comprehensive eutrophication grades were acquired by Equation (17), which was shown as follows:

(17) $$L^{k}=\sum_{s=0}^{q}J_{s}^{k}P_{s}$$

where $L^{k}$ is comprehensive eutrophication grade for the *k*-th lake, *q* is the number of lake’s possible eutrophication grade, $J_{s}^{k}$ is the possible eutrophication grade for the *k*-th lake, $P_{s}$ is the probabilities of each eutrophication grade, which was obtained by MC simulation of forecast variable $L^{k}$. With reference to the classification of Table 4, the comprehensive eutrophication grades of lakes were determined. The variables $L^{k}$ was defined as the predictive variables. The frequency distribution of results became convergent when *N* is large enough. 

**Figure 2:**
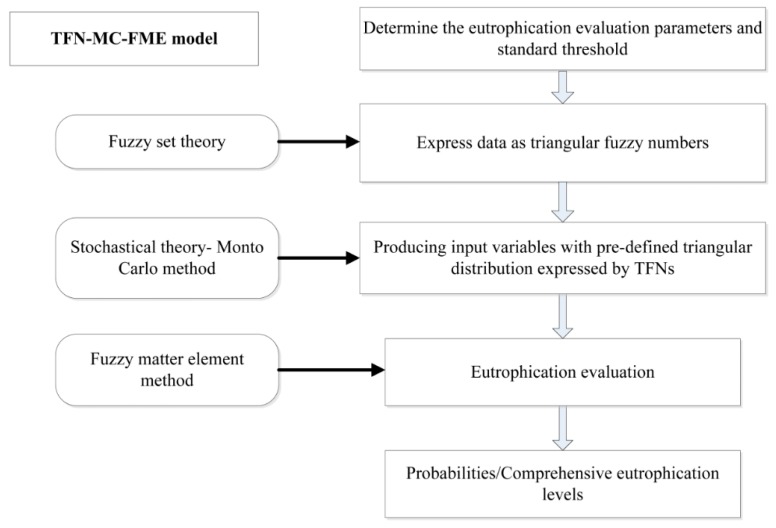
Flowchart of eutrophication assessment.

The flowchart of the hybrid TFN–MC–FME model is shown in Figure 2. 

## 3. Results and Discussions

### 3.1. Eutrophication Grade Evaluated by Hybrid TFN–MC–FME Model

**Figure 3:**
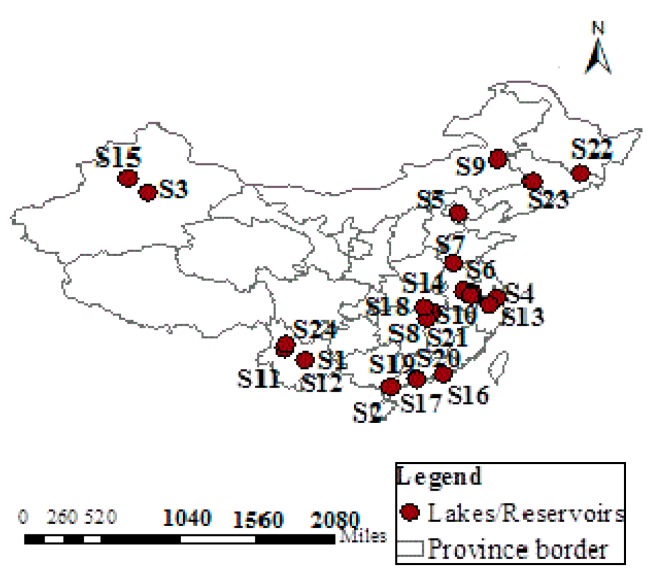
Locations of 24 typical lakes/reservoirs in China.

**Figure 4:**
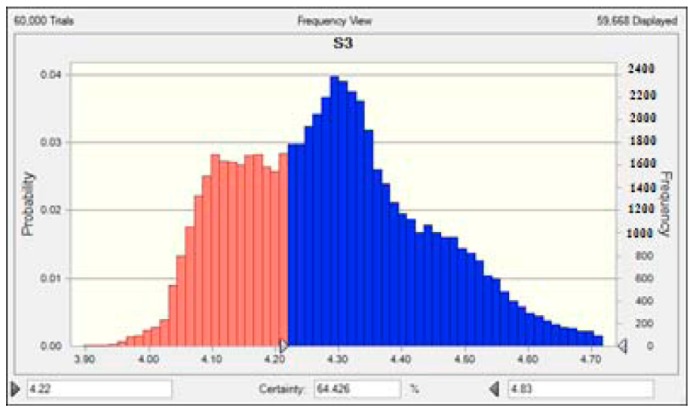
Simulation results of Bosten Lake (S3).

The statistics data of indicators for eutrophication evaluation of 24 lakes were expressed with TFN, given in Table 5 and Figure 3 [22,23]. Based on the developed methodology, the eutrophication statuses of 24 typical lakes in China were assessed. For each lake, 60,000 simulation instances were performed to obtain convergent results separately. Bosten Lake was selected to illustrate the typical simulation course of TFN–MC–FME model, shown in Figure 4. The simulation results are shown in Table 6. The threshold of each eutrophication grade in the Crystal Ball software was set according to the classification of eutrophication levels in Table 4 which was determined by non-integral eutrophication feature value. In addition, the non-integral eutrophication feature values for lakes were calculated by Equation (14), and the possible intervals of each eutrophication grade and their corresponding probabilities were obtained, shown in Table 7. Finally, the eutrophication grades of lakes were determined by Equation (17) by multiplying the probabilities of the eutrophication grades with the corresponding eutrophication grade, shown in Table 8. 

As shown in Table 7, the possible intervals and the corresponding probabilities for each lake were obtained. In many lakes, two possible intervals showed a significant probability, which indicate that the evaluation of eutrophication levels involved a great deal of uncertainties, especially for the lakes with small difference between two eutrophication levels, such as S4, S5, S14, and S15. 

As shown in Table 8, the comprehensive eutrophication levels were in the order of S12 = S20 > S17 > S19 > S18 > S21 > S16 > S23 > S14 > S15 > S13 > S11 > S9 > S10 > S8 = S22 > S7 > S6 > S5 > S4 > S3 > S24 > S2 > S1. With respect to the probabilities of the eutrophication grades in Table 7, Erhai Lake (S1), Gaoshan Lake (S2), Bosten Lake (S3), and Qionghai Lake (S24) have the greatest likelihood of being in grade IV. In addition, Gucheng Lake (S6), Nansi Lake (S7), Dali Lake (S9), Chao Lake (S10), Dianchi Lake (Outer sea) (S11), West Lake (S13), and Jingpo Lake (S22) have the greatest likelihood to be in the grade V. Moreover, Li Lake (S16), Dongshan Lake (S17), Moshui Lake (S18), Liwan Lake (S19), Xuanwu Lake (S21), and Nan Lake (S23) have the greatest likelihood to be in the grade VI. For Dianshan Lake (S4), the comprehensive eutrophication grade of Dianshan Lake is grade IV, and the probability of being in the grade IV is close to being in the grade V, which means that the lake has a worsening tendency to become grade V. For Yuqiao reservoir (S5), the comprehensive eutrophication grade is V, and the probability of being in the grade V approximates the probability of being in the grade IV, which indicates that the lake has an improving tendency from grade V to grade IV. The comprehensive eutrophication grades for Gantang Lake (S14) and Mogu Lake (S15) are grade V as well, and the probability of being in the grade V approximates the probability of being in the grade VI, which means that Gantang Lake (S14) and Mogu Lake (S15) have the worsening trend from grade V to grade VI. Specifically, for Ci Lake (S8), the probability of being in grade V is 100%, which means that although there is the uncertainty in data of Ci Lake, the eutrophication grade is V without exception. Similarly, for Dianchi Lake (Cao Sea) (S12) and Liuhua Lake (S20), the probability of being in grade VI is 100%, which indicates that the eutrophication grade is VI without exception. Therefore, compared with the determined method, the proposed TFN–MC–FME model can identify the developing trends of eutrophication in lakes and provide lake managers with more valuable information. For example, lake managers can get the variation intervals of eutrophication status, and find the seasons or zones in the lake that may be seriously threatened by eutrophication, which can help make scientific schemes and planning relate to lakes. 

### 3.2. Comparison with Other Approaches

The eutrophication evaluation of these 24 lakes was performed by researchers with methods of trophic level index method (TLI), back propagation neutral network (BNN), and projection pursuit (PP) method. The comparison of the proposed model with the three methods is shown in Table 9. For almost all lakes except for Erhai Lake (S1), Gaozhou Reservoir (S2), Bosten Lake (S3), Yuqiao Reservoir (S5), and Qionghai Lake (S24), the evaluation results were completely consistent with the other three methods, which proved the correctness of TFN–MC–FME model. However, for some lakes, there are some differences between the developed method and the deterministic method which used the average values of water quality data. For Erhai Lake (S1) and Gaozhou Reservoir (S2), the eutrophication grade is IV according to the results of developed method. However, for the results of the other deterministic methods, they were in grade III. For Bosten Lake (S3), the result obtained by the proposed method is grade IV, which is not consistent with the results obtained by PP method. In addition, For Yuqiao Reservoir (S5), the result obtained by proposed model is grade V, which is not the same as the result of TLI and BNN methods. For Qionghai Lake (S24), the result obtained by the proposed model is grade IV, which is not consistent with the result of TLI method. It can be explained that in the proposed model, the distributions of data were considered comprehensively, which leads to different results from other methods. In addition, the assessment results of TLI, BNN, and PP are based on the average values of indicators, while the assessment result of hybrid method is based on the triangular distribution of indicators, which leads to the difference between proposed model and other methods. When the monitoring data of indicators are asymmetrical with a great variation, the hybrid method is more useful than the other methods. Although for most of the lakes, the assessment results of hybrid method are consistent with the deterministic methods that seem to be easier to perform, the hybrid model can supply more information of eutrophication status including the probability distribution of eutrophication feature value shown in Table 6 and Table 7, and Figure 3. For example, for Erhai Lake (S1), Gaozhou Reservoir (S2), Bosten Lake (S3), and Dianshan Lake (S4), the eutrophication status is the same of IV. However, the comprehensive eutrophication values from Table 8 are 3.971, 3.986, 4.08, and 4.465, respectively, which indicated that the eutrophication degree increased in the order of S1 < S2 < S3 < S4. By stochastically simulating the data according to the distribution character of data, the possible eutrophication grades with the corresponding probabilities were obtained. Therefore, the occurrence of the accidental value that may affect the assessment result can be prevented. Since the hybrid model is based on the probability distribution, when the distributions of indicators cannot be obtained from the monitoring data correctly, the application of the hybrid method will be constrained. 

## 4. Conclusions

In this study, a method coupling the TFN method, MC approach, and FME model was proposed and applied to evaluate the eutrophication status of typical lakes in China. The results indicated that the model can be used to evaluate the eutrophication grade scientifically and objectively, and eutrophication grades in the studied typical lakes in China ranged from IV to VI, which means that the service function of these lakes will be unavailable except for taking measures to protect the water environment of lakes. With the developed model, the eutrophication trends of lakes can be obtained by the considering the possible intervals and corresponding probabilities. The uncertainties of data can be processed by the TFN method, the fuzziness of the evaluation method can be improved by the FME model, and the operation difficulty of TFN in the FME model can be solved by the MC approach. Therefore, the proposed method can also be applied to other evaluation fields involving uncertainty.

## Figures and Tables

**Table 1:** Comparison of eutrophication evaluation methods.

Approach	Examples	Advantages	Limitations
Multivariate statistical techniques	Including cluster analysis (CA), discriminant analysis (DA), principle component analysis/factor analysis (PCA/FA).	-Can solve randomness of monitored data -Reduce a complex data set to a considerably low dimension -Obtain the underlying patterns within the original data	-Require larger samples -Data are difficult to obtain
Comprehensive assessment method	Including fuzzy set theory based on fuzzy membership function, matter element model, etc.	-Reflected the fuzziness in the evaluation of the classification standard, evaluation class, and degree of eutrophication -Solve uncertainty	Weak to distinguish the adjacent characteristic indicators
Machine learning approaches	Including artificial neural networks (ANN), support vector machine, and random forests (RF), etc.	-Provide predictive models with good generalization abilities -Capture unknown patterns in the assessment process -Infer complex relationships without knowledge of a system	-Lacks to accurately analyze each performance index
Hybrid models	Including method combined with neuro fuzzy networks with factor analysis, cloud model considering randomness with fuzziness, etc.	Combine the advantages of different methods	-Complicated model structure

**Table 2:** Criteria of grading index for eutrophication of lake.

Rank	Chl-a (mg/m^3^)	COD_Mn_ (mg/L)	TP (mg/m^3^)	TN (mg/m^3^)	SD (m)
I	≤0.5	≤0.15	≤1	≤20	≥10
II	≤1	≤0.4	≤4	≤50	≥5
III	≤4	≤2.0	≤25	≤300	≥1.5
IV	≤10	≤4.0	≤50	≤500	≥1.0
V	≤64	≤10.0	≤200	≤2000	≥0.4
VI	>64	>10	>200	>2000	<0.4

Note: Chl-a, COD_Mn_, TP, TN, SD refer to chlorophyII-a, chemical oxygen demand, total phosphorus, total nitrogen (TN), and clarity (SD), respectively.

**Table 3:** Weights of indicators for eutrophication assessment.

Indicators	Entropy	Entropy Weight	AHP Weight	Entropy–AHP Weight *w*
Chl-a	2.97	0.12	0.46	0.32
COD_Mn_	4.54	0.21	0.15	0.19
TP	5.12	0.24	0.09	0.13
TN	4.85	0.23	0.05	0.077
SD	4.48	0.20	0.25	0.30

**Table 4:** Definition of eutrophication grade by non-integral eutrophication feature value *J.*

J	(1, 1.5]	(1.5, 2.5]	(2.5, 3.5]	(3.5, 4.5]	(4.5, 5.5]	(5.5, 6]
Grade	I	II	III	IV	V	VI

**Table 5:** Lower, expected and upper value of TFN approach for Lake (termed as A_1_, A_2_, and A_3_, respectively).

Sampling Sites	Chl-a (mg/m^3^)			COD_Mn_ (mg/L)			TP (mg/m^3^)			TN (mg/m^3^)			SD (m)		
	*A*_1_	*A*_2_	*A*_3_	*A*_1_	*A*_2_	*A*_3_	*A*_1_	*A*_2_	*A*_3_	*A*_1_	*A*_2_	*A*_3_	*A*_1_	*A*_2_	*A*_3_
Erhai Lake (S1)	0.49	1.86	3.00	1.70	3.09	3.24	4	22	40	160	246	465	1.22	2.77	3.45
Gaoshan Lake (S2)	0.28	1.49	5.24	0.56	1.47	3.28	17	46	86	187	358	652	1.08	1.72	2.54
Bosten Lake (S3)	1.74	3.52	6.59	2.85	5.96	11.08	12	23	41	457	932	1598	0.58	1.46	3.04
Dianshan Lake (S4)	1.35	3.00	9.34	1.76	2.87	4.98	6	29	50	408	1086	1732	0.19	0.67	1.47
Yuqiao reservoir (S5)	2.83	10.79	22.71	1.08	4.11	9.65	4	25	53	325	1220	2564	0.38	1.42	2.16
Gucheng Lake (S6)	0.54	4.99	8.32	0.95	2.75	4.58	12	52	118	598	2374	5462	0.05	0.28	0.59
Nansi Lake (S7)	0.28	3.77	8.76	2.58	6.96	11.49	63	194	432	1248	3201	6325	0.12	0.44	0.76
Ci Lake (S8)	3.68	14.47	42.36	0.87	3.74	7.64	26	77	186	350	1000	2512	0.10	0.36	0.64
Dali Lake (S9)	1.38	7.24	15.24	8.27	16.25	34.58	24	153	354	425	1671	3514	0.16	0.48	1.14
Chao Lake (S10)	3.85	11.80	31.65	2.56	4.01	9.86	36	115	364	546	1786	3256	0.05	0.28	0.62
Dianchi Lake(Outer sea) (S11)	16.52	44.43	85.36	2.58	7.11	14.56	36	108	328	357	1309	2658	0.19	0.49	0.87
Dianchi Lake (Cao Sea) (S12)	98.27	298.86	456.92	5.68	16.58	38.75	357	931	1456	685	15,273	24,365	0.06	0.23	0.42
West Lake (S13)	15.68	58.95	115.64	0.68	6.94	17.28	39	161	426	426	2478	2768	0.15	0.43	0.84
Gantang Lake (S14)	29.56	75.69	158.64	0.98	7.23	21.32	38	141	325	346	1417	2541	0.09	0.38	0.73
Mogu Lake (S15)	8.96	54.77	128.47	2.38	10.38	24.19	56	287	574	624	2206	4567	0.21	0.53	0.87
Li Lake (S16)	37.54	119.51	326.98	2.13	9.92	34.67	84	372	753	1524	3038	5367	0.16	0.34	0.62
Dongshan Lake (S17)	29.34	149.45	514.28	3.48	13.40	25.86	158	428	796	1645	5350	7658	0.08	0.22	0.43
Moshui Lake (S18)	48.37	153.59	358.69	2.49	13.51	38.62	95	232	467	7853	15,692	26,342	0.06	0.22	0.54
Liwan Lake (S19)	46.32	162.92	362.97	5.62	14.46	34.25	249	743	1124	2405	7337	11,246	0.13	0.31	0.64
Liuhua Lake (S20)	75.49	323.51	615.24	8.37	25.26	42.63	342	643	1024	3248	6777	9754	0.03	0.15	0.32
Xuanwu Lake (S21)	28.67	168.14	324.56	3.62	10.08	25.98	158	663	1247	1125	4073	7654	0.05	0.22	0.42
Jingpo Lake (S22)	0.98	4.96	14.35	1.67	5.96	24.37	88	316	647	324	1270	2485	0.26	0.73	1.08
Nan Lake (S23)	21.71	120.60	328.45	2.38	8.22	21.57	65	228	497	1028	2630	3782	0.06	0.22	0.41
Qionghai Lake (S24)	0.19	0.88	3.28	0.54	1.43	4.52	57	130	268	217	410	862	1.08	2.98	4.32

Note: 1. Chl-a, COD_Mn_, TP, TN, SD refer to ChlorophyII-a, chemical oxygen demand, total phosphorus, total nitrogen (TN), and clarity (SD), respectively. 2. The data for calculating A1, A2, and A3 were taken from literatures published in Chinese during period from 1993 to 2017.

**Table 6:** Non-integral eutrophication grade rank feature of each lake.

Cases	Minimum Values	Average Values	Maximum Values
Erhai Lake (S1)	3.33	3.77	4.13
Gaoshan Lake (S2)	3.32	3.89	4.45
Bosten Lake (S3)	3.85	4.24	4.85
Dianshan Lake (S4)	3.90	4.48	4.99
Yuqiao reservoir (S5)	3.56	4.55	5.19
Gucheng Lake (S6)	4.07	4.83	5.19
Nansi Lake (S7)	4.32	5.00	5.37
Ci Lake (S8)	4.56	5.16	5.44
Dali Lake (S9)	4.56	5.13	5.61
Chao Lake (S10)	4.81	5.26	5.56
Dianchi Lake(Outer sea) (S11)	4.87	5.26	5.68
Dianchi Lake (Cao Sea) (S12)	5.60	5.82	5.91
West Lake (S13)	4.82	5.36	5.82
Gantang Lake (S14)	4.93	5.48	5.83
Mogu Lake (S15)	4.96	5.44	5.83
Li Lake (S16)	5.21	5.69	5.87
Dongshan Lake (S17)	5.37	5.78	5.90
Moshui Lake (S18)	5.28	5.75	5.90
Liwan Lake (S19)	5.34	5.74	5.89
Liuhua Lake (S20)	5.69	5.87	5.91
Xuanwu Lake (S21)	5.40	5.77	5.91
Jingpo Lake (S22)	4.39	5.02	5.53
Nan Lake (S23)	5.16	5.69	5.90
Qionghai Lake (S24)	3.41	3.85	4.47

**Table 7:** Probable intervals of non-integral eutrophication grade, corresponding probabilities, and comprehensive eutrophication status.

Cases	Possible Intervals of Non-Integral Eutrophication Feature Value Eutrophication Grade	Probability (%)	Eutrophication Status
Erhai Lake (S1)	[3.30, 3.50]	2.87	III
	[3.50, 4.16]	97.13	IV
Gaoshan Lake (S2)	[3.32, 3.50]	1.37	III
	[3.50, 4.45]	98.63	IV
Bosten Lake (S3)	[3.85, 4.50]	92.05	IV
	[4.50, 4.85]	7.95	V
Dianshan Lake (S4)	[3.90, 4.50]	53.49	IV
	[4.50, 4.99]	46.51	V
Yuqiao reservoir (S5)	[3.56, 4.50]	41.82	IV
	[4.50, 5.19]	58.18	V
Gucheng Lake (S6)	[4.07, 4.50]	1.09	IV
	[4.50. 5.19]	98.91	V
Nansi Lake (S7)	[4.32, 4.50]	0.14	IV
	[4.50, 5.37]	99.86	V
Ci Lake (S8)	[4.56, 5.44]	100	V
Dali Lake (S9)	[4.56, 5.50]	99.63	V
	[5.50, 5.61]	0.37	VI
Chao Lake (S10)	[4.81, 5.50]	99.66	V
	[5.50, 5.56]	0.34	VI
Dianchi Lake (Outer sea) (S11)	[4.87, 5.50]	99.26	V
	[5.50, 5.68]	0.74	VI
Dianchi Lake (Cao Sea) (S12)	[5.60, 5.91]	100	VI
West Lake (S13)	[4.82, 5.50]	85.66	V
	[5.50, 5.82]	14.34	VI
Gantang Lake (S14)	[4.93, 5.50]	54.03	V
	[5.50, 5.83]	45.97	VI
Mogu Lake (S15)	[4.96, 5.50]	69.41	V
	[5.50, 5.83]	30.59	VI
Li Lake (S16)	[5.21, 5.50]	3.50	V
	[5.50, 5.87]	96.50	VI
Dongshan Lake (S17)	[5.37, 5.50]	0.19	V
	[5.50, 5.90]	99.81	VI
Moshui Lake (S18)	[5.28, 5.50]	0.59	V
	[5.50, 5.90]	99.41	VI
Liwan Lake (S19)	[5.34, 5.50]	0.29	V
	[5.50, 5.89]	99.71	VI
Liuhua Lake (S20)	[5.69, 5.91]	100	VI
Xuanwu Lake (S21)	[5.40, 5.50]	0.04	V
	[5.50, 5.91]	99.61	VI
Jingpo Lake (S22)	[4.39, 4.50]	0.04	IV
	[4.50, 5.50]	99.94	V
	[5.50, 5.53]	0.02	VI
Nan Lake (S23)	[5.16, 5.50]	4.13	V
	[5.50, 5.90]	95.87	VI
Qionghai Lake (S24)	[3.41, 3.50]	1.31	III
	[3.50, 4.47]	98.69	IV

**Table 8:** Comprehensive eutrophication values of lakes.

Cases	Comprehensive Eutrophication Values	Final Eutrophication Grades
Erhai Lake (S1)	3.971	IV
Gaoshan Lake (S2)	3.986	IV
Bosten Lake (S3)	4.080	IV
Dianshan Lake (S4)	4.465	IV
Yuqiao reservoir (S5)	4.582	V
Gucheng Lake (S6)	4.989	V
Nansi Lake (S7)	4.999	V
Ci Lake (S8)	5.000	V
Dali Lake (S9)	5.004	V
Chao Lake (S10)	5.003	V
Dianchi Lake(Outer sea) (S11)	5.007	V
Dianchi Lake (Cao Sea) (S12)	6.000	VI
West Lake (S13)	5.143	V
Gantang Lake (S14)	5.460	V
Mogu Lake (S15)	5.306	V
Li Lake (S16)	5.965	VI
Dongshan Lake (S17)	5.998	VI
Moshui Lake (S18)	5.994	VI
Liwan Lake (S19)	5.997	VI
Liuhua Lake (S20)	6.000	VI
Xuanwu Lake (S21)	5.979	VI
Jingpo Lake (S22)	5.000	V
Nan Lake (S23)	5.959	VI
Qionghai Lake (S24)	3.987	IV

**Table 9:** Comparison of eutrophication grade between the proposed TFN–MC–FME model and the other relevant methods.

Cases	Hybrid Method	Trophic Level Index (TLI) [24]	Back Propagation Neutral Network [25]	Projection Pursuit Method [26]
Erhai Lake (S1)	IV	III	III	III
Gaozhou Reservoir (S2)	IV	III	III	III
Bosten Lake (S3)	IV	IV	IV	V
Dianshan Lake (S4)	IV	IV	IV	IV
Yuqiao reservoir (S5)	V	IV	IV	V
Gucheng Lake (S6)	V	V	V	V
Nansi Lake (S7)	V	V	V	V
Ci Lake (S8)	V	V	V	V
Dali Lake (S9)	V	V	V	V
Chao Lake (S10)	V	V	V	V
Dianchi Lake(Outer sea) (S11)	V	V	V	V
Dianchi Lake (Cao Sea) (S12)	VI	VI	VI	VI
West Lake (S13)	V	V	V	V
Gantang Lake (S14)	V	V	V	V
Mogu Lake (S15)	V	V	V	VI
Li Lake (S16)	VI	VI	VI	VI
Dongshan Lake (S17)	VI	VI	VI	VI
Moshui Lake (S18)	VI	VI	VI	VI
Liwan Lake (S19)	VI	VI	VI	VI
Liuhua Lake (S20)	VI	VI	VI	VI
Xuanwu Lake (S21)	VI	VI	VI	VI
Jingpo Lake (S22)	V	V	V	V
Nan Lake (S23)	VI	VI	VI	VI
Qionghai Lake (S24)	IV	III	VI	IV
